# Supplementary material for: Complexome profiling of the Chlamydomonas psb28 mutant reveals TEF5 as an early PSII assembly factor
Source: Plant Cell. 2025 Mar 17;37(6):koaf055. doi: 10.1093/plcell/koaf055 (PMC12164590; doi:10.1093/plcell/koaf055)
Supplement: koaf055_Supplementary_Data [file koaf055_supplementary_data.zip › psb28_interactive.zip!/plots/intensity/qualityPlots/peptides/_Cre08.g368700.t1.1.html]

### Cre08.g368700.t1.1

Trivial name:   
SeqCoverage [median]: 10.40  
Mass: 54.80 kDa  
  
 

| Sequence | MissedCleavage | Mass [Da] | wtIntensity | muIntensity | Score |
| --- | --- | --- | --- | --- | --- |
| FAGVKPSTFAGIR | 1 | 1349.75 | 698.59 | 6318.18 | 55.45 |
| HSAVVLAPK | 0 | 920.54 | 982.78 | 3461.98 | 93.14 |
| KGGAGSASAAEAAALSFR | 1 | 1620.82 | 0.00 | 1869.15 | 45.82 |
| TFSGPYIGSTLSDR | 0 | 1499.73 | 0.00 | 671.28 | 73.85 |
|  |  |  | 1681.4 | 12320.6 |  |
